# Assessing the influence of kernel selection on chest computed tomography image quality across varying dose levels using TrueFidelity reconstruction

**DOI:** 10.1093/rpd/ncaf143

**Published:** 2026-03-13

**Authors:** Eleftheria Gianko, Micael Oliveira Diniz, Walter Cifuentes Ramirez, Rauni Rossi Norrlund, Åse A Johnsson, Magnus Båth, Angelica Svalkvist

**Affiliations:** Department of Medical Radiation Sciences, Institute of Clinical Sciences, Sahlgrenska Academy, University of Gothenburg, Gothenburg, Sweden; Region Västra Götaland, Sahlgrenska University Hospital, Department of Biomedical Engineering and Medical Physics, Gothenburg Sweden; Department of Radiology, Institute of Clinical Sciences, Sahlgrenska Academy, University of Gothenburg, Gothenburg, Sweden; Region Västra Götaland, Sahlgrenska University Hospital, Department of Radiology, Gothenburg, Sweden; Region Västra Götaland, Sahlgrenska University Hospital, Department of Radiology, Gothenburg, Sweden; Department of Radiology, Institute of Clinical Sciences, Sahlgrenska Academy, University of Gothenburg, Gothenburg, Sweden; Region Västra Götaland, Sahlgrenska University Hospital, Department of Radiology, Gothenburg, Sweden; Department of Radiology, Institute of Clinical Sciences, Sahlgrenska Academy, University of Gothenburg, Gothenburg, Sweden; Region Västra Götaland, Sahlgrenska University Hospital, Department of Radiology, Gothenburg, Sweden; Department of Medical Radiation Sciences, Institute of Clinical Sciences, Sahlgrenska Academy, University of Gothenburg, Gothenburg, Sweden; Region Västra Götaland, Sahlgrenska University Hospital, Department of Biomedical Engineering and Medical Physics, Gothenburg Sweden; Department of Medical Radiation Sciences, Institute of Clinical Sciences, Sahlgrenska Academy, University of Gothenburg, Gothenburg, Sweden; Region Västra Götaland, Sahlgrenska University Hospital, Department of Biomedical Engineering and Medical Physics, Gothenburg Sweden

## Abstract

Deep learning image reconstruction (DLIR) utilizes neural networks to generate high-quality computed tomography (CT) images. One commercially available DLIR software is TrueFidelity from GE Healthcare. The Standard kernel was the only available reconstruction kernel previously, but recently other kernels, including the Lung kernel, have been introduced by GE. This study aimed to evaluate the image quality of chest CT scans acquired at full-dose (FD, 2.5 mSv) and ultra-low-dose (ULD, 0.05 mSv) when reconstructed using TrueFidelity with both Standard and Lung kernels. Twenty-five patients underwent chest CT scans at Sahlgrenska University Hospital. The images were reconstructed and then evaluated by four radiologists in two different studies, one including ULD CT axial images and the other one the FDCT. Visual Grading Characteristics (VGC) analysis was applied, using the Standard kernel as reference and the area under the VGC curve (AUC_VGC_) for comparison. At FD, the Standard kernel yielded better results regarding the visualization of six structures and the general image quality. However, in ULD scans, the differences between kernels were not statistically significant. The FD images were mostly rated as acceptable, while ULD images were often rated as probably acceptable or unacceptable, especially for emphysema assessment. Overall, TrueFidelity seems to perform better with the Standard kernel than with the Lung kernel in FD protocols, but no reliable conclusions can be drawn for the ULD protocol.

## Introduction

Since its inception, computed tomography (CT) has undergone continuous advancements, establishing itself as an essential tool in medical imaging. It is the most commonly used cross-sectional imaging technique nowadays. Modern CT scanners are equipped with large detector arrays and rapid tube rotation. These advancements enable whole-body examinations within seconds, thus providing rapid and detailed diagnostic information [1–3]. Due to its efficiency and diagnostic capabilities, CT is extensively used for disease screening, diagnosis, treatment monitoring, and follow-up assessments, contributing to a steady rise in the annual number of CT examinations performed [1, 4].

Over the past few decades, the growing popularity of CT has raised concerns about human exposure to ionizing radiation. Optimization strategies such as the “as low as reasonably achievable” principle have resulted in ongoing technological improvements aimed at reducing radiation exposure [1, 5]. However, the fundamental trade-off persists between image quality and radiation dose, where the visibility of subtle image features and diagnostic accuracy may get affected by a reduced radiation exposure [6]. Consequently, current advancements in CT imaging technology have been primarily focused on establishing methods for improving image quality and thus potentially reducing radiation exposure [7]. Regarding radiation dose, low-dose (LD) CT and ultra-low-dose (ULD) CT, the latter with a dose level comparable to conventional X-ray (CXR), serve as promising alternatives especially for medical examinations such as chest CT [3]. In this article, the term ULD is used for a CT protocol with radiation dose equal to 0.05 mSv for standard-sized patients, which is in the order of frontal and lateral chest radiographs conducted at Sahlgrenska University Hospital [8].

Image reconstruction techniques have been a major focus in recent years aiming to improve image quality while reducing noise in LD conditions [6]. The reconstruction algorithm employed, which is the mathematical process to create the CT image from acquired raw data, plays a critical role in CT image quality, directly influencing spatial resolution, noise levels, and artefact suppression [1, 9]. Up until present, the primary reconstruction method has been filtered back projection (FBP) [10]. However, compared to newer reconstruction techniques, FBP requires relatively high radiation doses to achieve acceptable image noise levels [2, 11]. To address this issue, iterative reconstruction (IR) techniques were introduced, making use of statistical models to differentiate true signals from noise, thereby enabling lower radiation doses while maintaining image quality [12]. Despite its advantages, IR has been criticized for producing images with an artificial or “plastic” appearance due to texture alterations, which may affect radiologists' interpretation and diagnostic confidence [11, 12].

Deep learning-based image reconstruction techniques represent a novel approach that utilizes deep neural networks (DNNs) to generate high-quality images with reduced noise, while preserving image texture comparable to FBP and IR [7, 11, 13, 14]. Unlike conventional reconstruction methods, deep learning-based CT reconstruction can enhance image quality without increasing patient radiation dose or prolonging reconstruction time making it an effective solution for CT at low doses [2, 15]. Deep learning image reconstruction (DLIR) models, typically trained on extensive low-dose imaging datasets [16], are capable of effective noise suppression and maintenance of clinically relevant anatomical structures [7, 16]. Preserving diagnostic accuracy while minimizing radiation exposure is of utmost importance, especially for examinations that only require LD CT settings [14, 16].

One of the earliest DLIR algorithms was introduced by GE Healthcare in 2019 and it is called TrueFidelity (GE Healthcare, Chicago, IL) [11]. TrueFidelity [11] was designed to enhance image quality by training DNN using high-quality FBP images from both phantoms and patient data. These images were considered as ground truth. The training process of the DLIR engine involved three stages: training, validation, and testing. During training, the engine received input data consisting of LD, high-noise images and compared them to corresponding high-dose, low-noise images. The difference between the generated images and the ground truth was measured in terms of noise, texture, resolution, and other metrics. The engine then fine-tuned its parameters through backpropagation to minimize the difference. This iterative process was repeated with thousands of training datasets until the output images closely matched the ground truth under various realistic conditions. Once training was complete, the DLIR engine underwent extensive validation and testing to ensure accuracy and robustness. After successful validation, the trained model was deployed in clinical environments to reconstruct high-quality CT images [11]. The DLIR engine offers three selectable reconstruction strength levels (low, medium, high) to balance noise reduction according to clinical needs. Different kernel options have recently become available in TrueFidelity and since this study focused on chest imaging, a comparison was made between the Standard and the Lung kernel.

In addition to the reconstruction method, the selection of the reconstruction kernel significantly influences image appearance. Kernels are crucial to determining the smoothness/sharpness of a CT image and therefore influence the balance between image detail and the level of noise in the image [17, 18]. They function as convolution algorithms, modifying the frequency content in projection data and impacting spatial resolution and noise levels. There are various reconstruction kernels available to better capture and enhance the characteristics of CT images. In chest CT, images are commonly reconstructed using two different kernels, a smooth kernel and a sharp kernel [19]. Smooth kernels are usually optimized for soft tissues, producing smoother images with lower noise but reduced spatial resolution as well. In contrast, sharp kernels enhance high-frequency content and preserve fine anatomical details, such as bone structures, though they inherently increase image noise [20]. For the GE Revolution APEX CT scanner (GE Healthcare, Chicago, IL), the Lung kernel is sharper than the Standard kernel, yielding higher spatial resolution but also increasing image noise [1]. For the reasons mentioned above, in the clinical routine at our hospital, two reconstructed image series are commonly created, one with the Standard kernel and one with the Lung kernel so that the radiologists could be able to examine all regions of the lung. While previous studies have demonstrated that the DLIR algorithm developed by GE Healthcare outperforms Adaptive Statistical Iterative Reconstruction (ASIR-V, GE Healthcare, Chicago, IL) in terms of image quality [1, 2, 6, 15, 21], there is limited data on the interaction between different kernels and DLIR across various CT examinations. Thus, investigating the combined effect of DLIR and different reconstruction kernels is essential for optimizing CT protocols to enhance image quality and diagnostic accuracy.

The primary object of this study was to assess and compare the image quality of chest CT examinations reconstructed using DLIR with both Standard and Lung kernel, across different dose levels (FD: 2.5 mSv, ULD: 0.05 mSv).

## Materials and methods

### Study patients

This study involved 25 patients who underwent chest CT scans without intravenous (IV) contrast at Sahlgrenska University Hospital between April 2021 and September 2023. The clinical questions included lung nodules, emphysema, and fibrosis. Details about the patient's gender, age, weight, and height were documented during the examination. The present study included eleven females and fourteen males, aged between 51 and 88 y. The body mass index ranged from 18.7 to 43.8 kg m^−2^. The Swedish Ethical Review Authority approved this study (reference number 2020-00581), and all participants gave written informed consent.

### Image data acquisition and reconstruction

The study was conducted using the GE Revolution APEX CT scanner (GE Healthcare, Chicago, IL), installed at the Sahlgrenska University Hospital. At the time of the study, the only available kernel for TrueFidelity reconstruction at our institution was the Standard kernel. However, for this study, raw data from the examination were extracted and taken to another hospital in the region (Skaraborg hospital), where TrueFidelity with Lung kernel was available.

The imaging procedure began with acquiring two scout images, after which a spiral CT scan was performed using a clinical FD protocol with an effective dose of 2.5 mSv for a standard-sized patient. This is also the standard imaging protocol used in the hospital. As part of the study, each patient also underwent an additional spiral scan using a ULD protocol. The ULD protocol was specifically tailored to provide an effective dose of around 0.05 mSv for a standard-sized patient, similar to the dose received from a CXR examination (including both frontal and lateral projections) at the hospital [8].

Images were acquired using spiral scans with a large body field-of-view and 80 mm collimation. For FD scans, parameters included a 0.5 s rotation time, pitch of 0.992, 0.625 mm slice thickness, 120 kV, and a variable mA range of 80–560, with organ dose modulation applied. In contrast, ULD scans used a faster rotation time of 0.28 s, a higher pitch of 1.531, thicker slices at 1.25 mm, 100 kV, and a tube current range of 10–15 mA, with dose modulation applied. The noise index was set at 31 for FD and 85 for ULD. After acquisition, the raw data were reconstructed with a slice thickness of 0.625 mm, which was used for the image quality assessment in both protocols. For each protocol, two different reconstructions were performed: DLIR-H with the Standard kernel and DLIR-H with the Lung kernel.

### Subjective image quality assessment

Two observer performance studies were created, one including the ULD CT images (Standard and Lung kernel) and the other one the FD CT images (Standard and Lung kernel). In both studies, only the axial view of the images was shown to the observers, in a randomized order for each individual. Four thoracic radiologists, with experience ranging from 3 to 25 y, participated in the study and evaluated image quality based on specific questions related to the reproduction of anatomical structures and overall image quality. The majority of the image quality questions (Qs) used in the study (Q1–Q5 in Table 1) were derived from image quality criteria established for chest CT examinations. These criteria were developed through a Delphi process [22] involving experts from five different institutions, as part of work package 2 of the European research project MEDIRAD—Implications of Medical Low Dose Radiation Exposure [23]. An additional criterion related to the visual reproduction of lymph node 4R (Q6 in Table 1) was added. The same set of questions was previously used in a comparative study by Svalkvist *et al*. [1] which assessed chest CT image quality using TrueFidelity (Standard kernel) and Adaptive Statistical Iterative Reconstruction (ASIR-V, Lung kernel). A summary of the questions posed to the observers is provided in Table 1.

**Table 1:** Image quality questions used for the observer performance study.

**Image quality questions**	
**Reproduction of anatomical structures**	**Answer alternatives**
Q1. Clear reproduction of the major fissure of the left lung (right if left is not visible)	1. Confident that the criterion is fulfilled 2. Somewhat confident that the criterion is fulfilled 3. I do not know if the criterion is fulfilled or not 4. Somewhat confident that the criterion is not fulfilled 5. Confident that the criterion is not fulfilled
Q2. Clear reproduction of B1: 3 subdivisions on axial plane of the apical bronchus of the right upper lobe (left upper lobe if right is not visible)	
Q3. Clear reproduction of A6: 4 divisions on axial plane of right apical pulmonary artery of the right lower lobe (left lower lobe if right one is not visible)	
Q4. Clear reproduction of B6: 3 divisions on axial plane of the apical bronchus of the right lower lobe (left lower lobe if right one is not visible)	
Q5. Clear reproduction of the right inferior pulmonary vein (RIPV): 3 divisions on axial plane (left if right one is not available)	
Q6. Clear reproduction of lymph node 4R (lymph node between VCS and trachea)	
**General image quality**	**Answer alternatives**
Q7. Image quality acceptable for diagnosis of pulmonary nodules?	A. Fully acceptable B. Probably acceptable C. Unacceptable
Q8. Image quality acceptable for diagnosis of fibrosis?	
Q9. Image quality acceptable for diagnosis of emphysema?	
Q10. Image quality acceptable for diagnosis of mediastinal inflammation (fat stranding)?	

The fulfilment of each criterion was rated on a five-point Likert scale, ranging from “Confident that the criterion is fulfilled” to “Confident that the criterion is not fulfilled.” Furthermore, the evaluation also included questions regarding the acceptability of the overall image quality for diagnosing common medical conditions (pulmonary nodules, fibrosis, emphysema, and mediastinal inflammation). The acceptability was rated on a three-level scale: “fully acceptable,” “probably acceptable,” and “unacceptable.”

The study was conducted using the ViewDEX 3.0 software (Viewer for Digital Evaluation of X-ray Images) [24–26], a tool specifically designed for observer performance studies. During the evaluation process, the observers were permitted to adjust the window width and level, as well as to zoom and pan the images. The radiologists were instructed to complete the study including the ULD images before proceeding with the study including the FD images.

### Statistical analysis of the results

The data were statistically analyzed using Visual Grading Characteristics (VGC) analysis [27], a non-parametric rank-invariant method designed to compare image quality between two different imaging modalities or protocols. In a VGC study, the observer is required to assess their confidence regarding the fulfillment of predefined image quality criteria using a multi-step rating scale. The technique compares the ratings for two distinct imaging protocols, in this case, CT images reconstructed using a Standard kernel (reference protocol) and those reconstructed with a Lung kernel (test protocol). The object is to evaluate how well each protocol satisfies the specified quality criteria, based on the observers' subjective assessments.

The ratings from the observers are subsequently plotted on a VGC curve, which illustrates the relationship between the proportions of fulfilled image quality criteria for both protocols at varying confidence thresholds. The area under the VGC curve $\left({\mathrm{AUC}}_{\mathrm{VGC}}\right)$serves as the primary metric for comparing the image quality of the two protocols. An ${\mathrm{AUC}}_{\mathrm{VGC}}>0.5$ indicates that the test protocol better meets the quality criteria, whereas an ${\mathrm{AUC}}_{\mathrm{VGC}}<0.5$ suggests that the test protocol is rated lower in terms of image quality. If the CI of the ${\mathrm{AUC}}_{\mathrm{VGC}}$ includes $0.5$, no statistically significant difference between the two protocols can be demonstrated, and the null hypothesis (H0) that the two protocols are equal cannot be rejected. This outcome may be due, for example, to the limited number of cases included in the study or to differences in image quality that are too small to be detected.

The VGC Analyzer software [28, 29] was employed to conduct the statistical analysis of visual grading data. The software calculates the ${\mathrm{AUC}}_{\mathrm{VGC}}$ through the trapezoidal rule and binormal curve fitting method. In the present study, the AUC was estimated utilizing the trapezoidal rule for curve approximation. The software is also capable of handling both paired and unpaired data, making it versatile for different study designs. In the present study the data were handled as paired since data from the same group of patients were reconstructed with different reconstruction kernels. Statistical analysis can be performed both for the fixed-reader situation and the random-reader situation as well. To determine the 95 per cent confidence interval (CI) of the AUC_VGC,_ the bootstrapping resampling technique is utilized. In the fixed-reader approach, data from all original observers are incorporated into the resampling process whereas in the random-reader approach a bootstrapping of observers is applied. Given the limited number of observers (*n* = 4), the fixed-reader approach was chosen for the analysis in the present study. Furthermore, permutation resampling is utilized to compute the *P*-value, which assesses the null hypothesis that the two protocols are equivalent.

## Results

**Figure 1:**
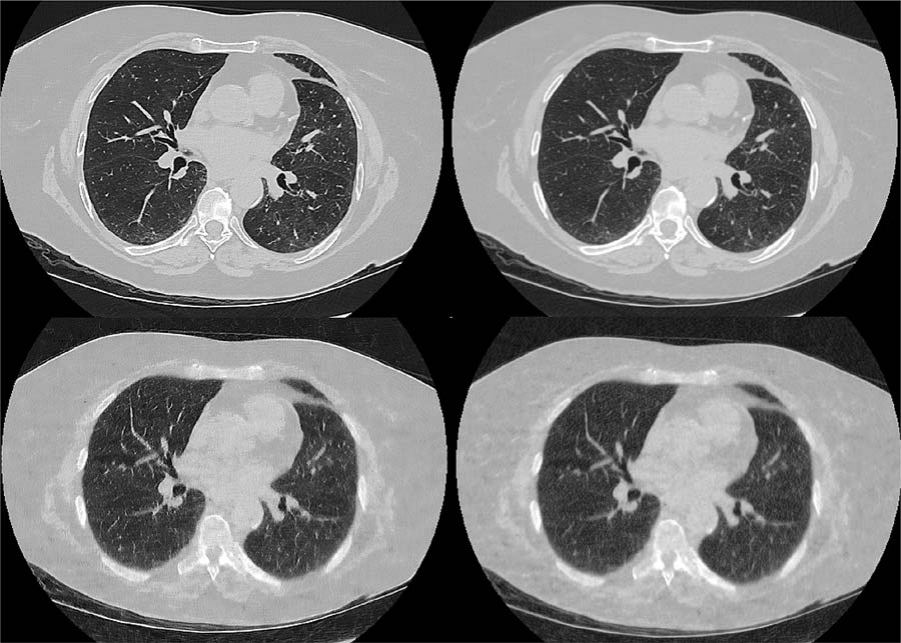
Axial CT images from the same patient acquired under full-dose (top row) and ultra-low-dose (bottom row) protocols, reconstructed using two reconstruction kernels: DLIR-H Lung kernel (left) and DLIR-H Standard kernel (right).

In Fig. 1, axial CT images are shown from examinations performed using the FD and ULD protocols, reconstructed using both DLIR-H with a Lung kernel and DLIR-H with a Standard kernel. The FD protocol at the top row represents images with superior image quality due to reduced noise and clearer visualization of anatomical details. When comparing the reconstructions with the Lung kernel to those with the Standard, a clear difference can be observed in all the images for both protocols regarding the noise presence as well as the edge enhancement in the lung kernel.

**Figure 2:**
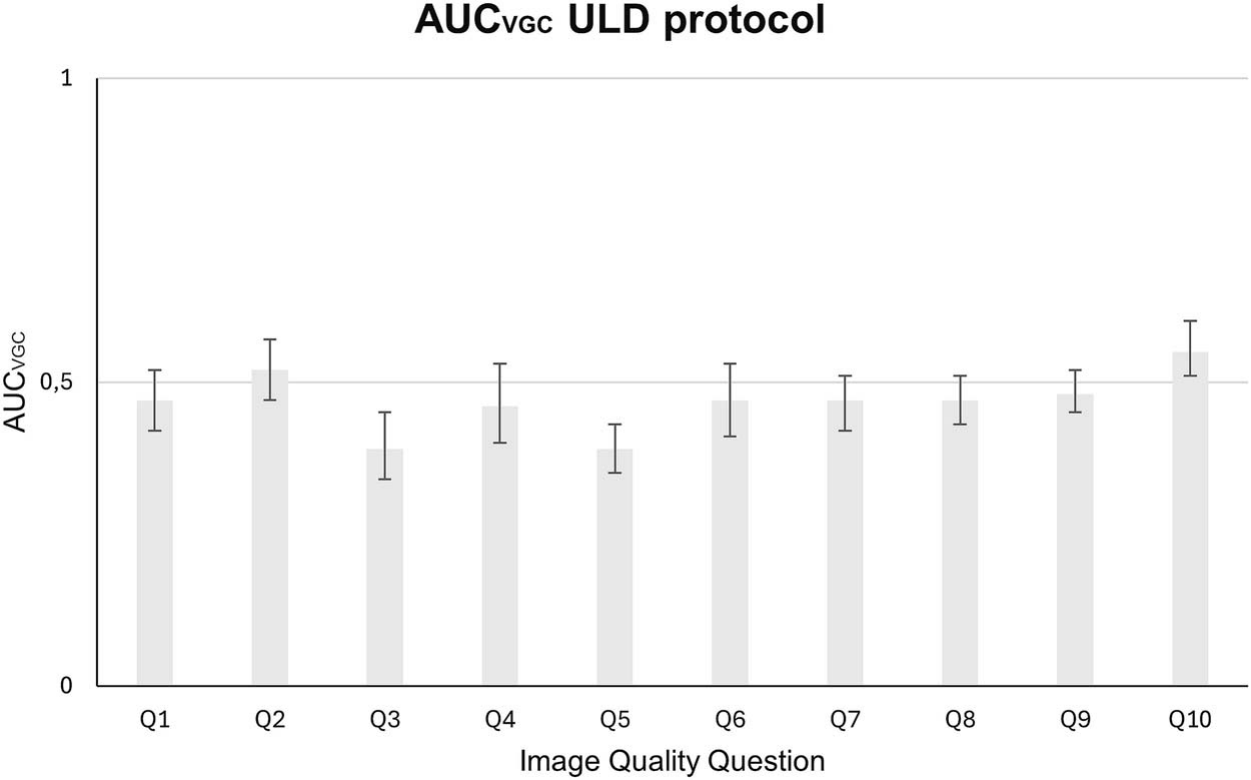
The resulting AUC_VGC_, along with its 95 per cent confidence interval, for the examinations conducted using the ultra-low-dose protocol, using TrueFidelity with the Standard kernel as the reference condition and TrueFidelity with the Lung kernel as the test condition.

**Figure 3:**
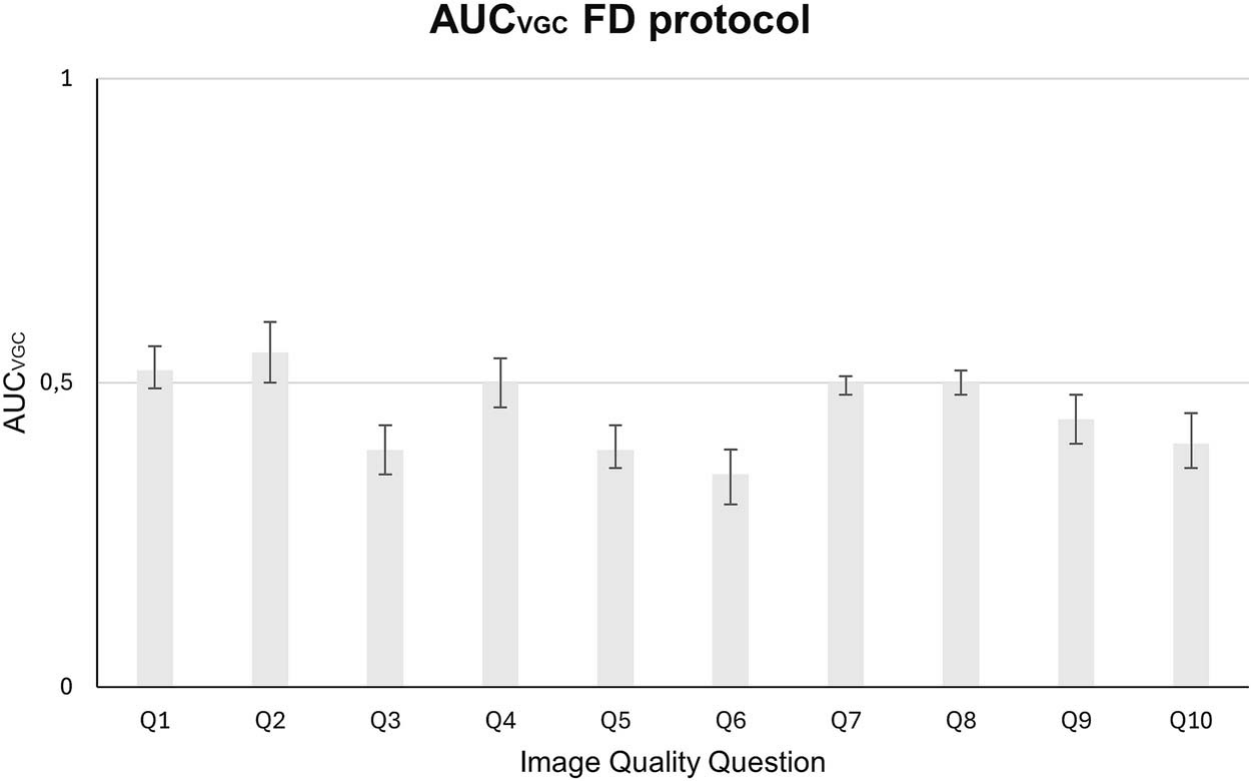
The resulting AUC_VGC_, along with its 95 per cent confidence interval, for the examinations conducted using the full-dose protocol, using TrueFidelity with the Standard kernel as the reference condition and TrueFidelity with the Lung kernel as the test condition.

The results of the VGC analysis, presented in Figs 2 and 3, display the ${\mathrm{AUC}}_{\mathrm{VGC}}$ values along with their 95 per cent CIs. The analysis was conducted on observer ratings covering two main categories: reproduction of anatomical structures (Q1–Q6) and overall image quality (Q7–Q10).

### Reproduction of anatomical structures

Regarding the questions that tried to evaluate which of the two different reconstruction kernels yielded better results in the reproduction of different anatomical structures, there were both similarities and differences for the two dose protocols. For the Q1, Q2, and Q4 questions, there was no statistically significant difference between the two kernels in either protocol. However, both the FD and the ULD protocol examinations reconstructed with the Standard kernel were rated significantly higher for Questions Q3 and Q5 that are related to the pulmonary artery and the pulmonary vein, respectively. In Q6, which evaluates the visibility of lymph node 4R (located between the VCS and trachea), the Standard kernel yielded significantly better results only in the FD protocol. No statistically significant difference was observed for the ULD protocol.

### General image quality

In terms of overall diagnostic acceptability of the images for common medical conditions (Q7–Q10), no significant differences were detected between the two kernels in Q7 and Q8 for either protocol. These questions concern the diagnostic acceptability for pulmonary nodules and fibrosis, respectively. Regarding the Q9 related to the diagnosis of emphysema, there was a different behavior observed between the two protocols. While for the FD protocol the Standard kernel yielded statistically significantly better results, for the ULD protocol no kernel showed statistically significant differences. A different behavior between the two protocols was also observed for Q10, which concerns the diagnosis of mediastinal inflammation. In the FD protocol, once again, there was a statistically significant preference for the Standard kernel. In contrast, for the ULD protocol, CT images reconstructed with the Lung kernel yielded statistically significantly higher results.

**Figure 4:**
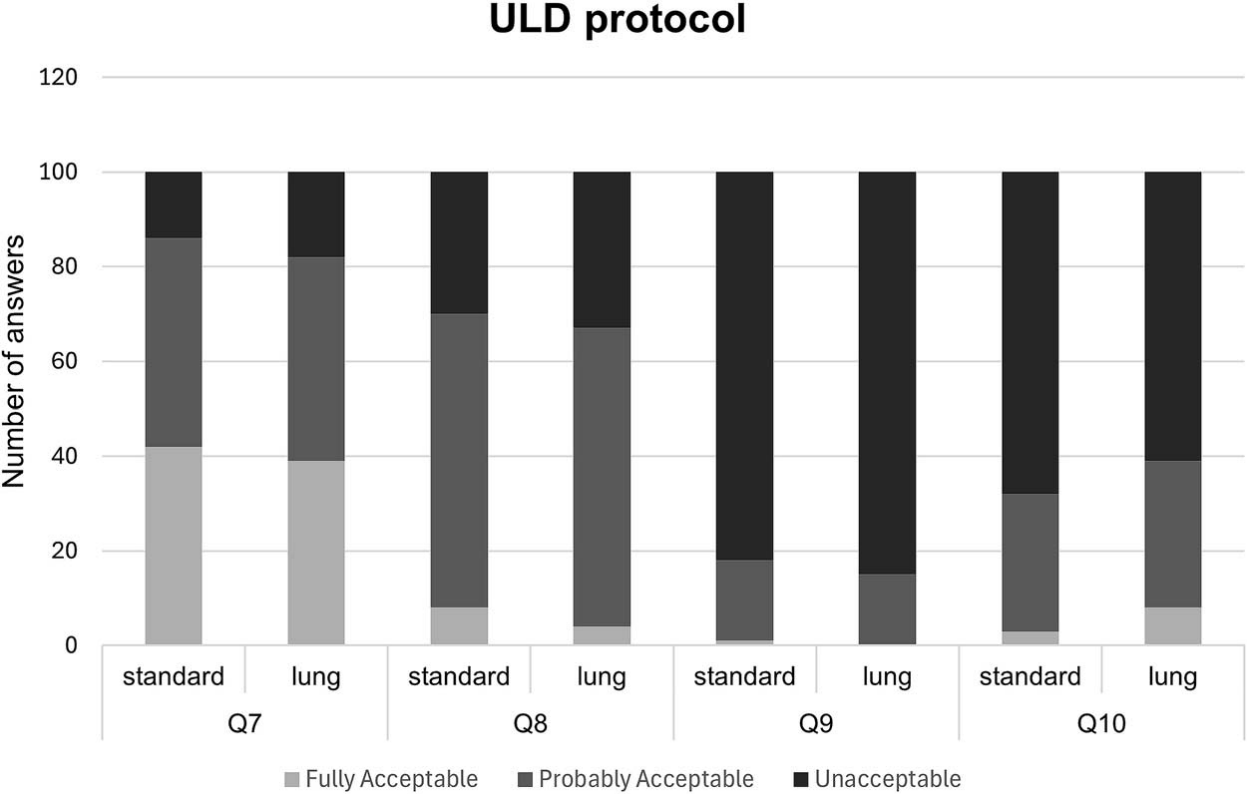
Ratings for Questions 7–10 about overall image quality in ultra-low dose scans for each answer category (fully acceptable, probably acceptable, unacceptable) pooled for all observers.

**Figure 5:**
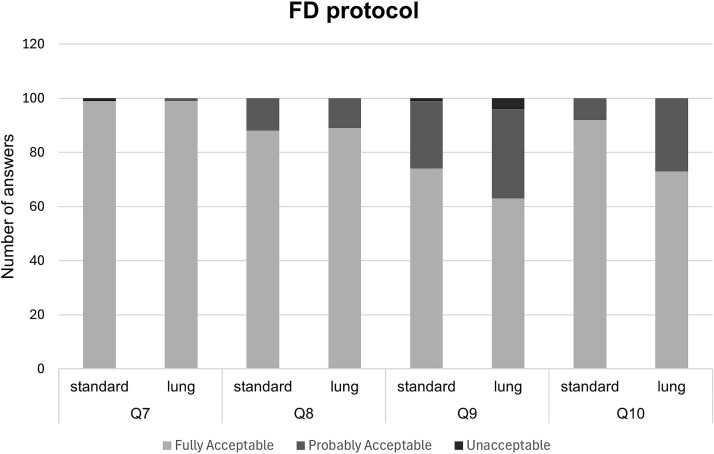
Ratings for Questions 7–10 about overall image quality in full-dose scans for each answer category (fully acceptable, probably acceptable, unacceptable) pooled for all observers.

Although the VGC analysis provided a comparison between the two kernels, it did not reveal whether the overall image quality for diagnosing various conditions was considered acceptable. Figures 4 and 5 present the distribution of responses (fully acceptable, probably acceptable, and unacceptable) for the general image quality questions (Q7–Q10), pooled for all observers.

A clear difference was observed between the two dose protocols across all evaluated questions. For the ULD protocol, more than 60 per cent of the images in each question received ratings of “probably acceptable” or “unacceptable,” suggesting that the ULD protocol may impact diagnostic findings. Especially in Q9 regarding the diagnosis of emphysema, 70 per cent of the images were considered to be unacceptable. In contrast, for the FD protocol, the majority of images were rated as acceptable for diagnostic purposes across both reconstruction kernels. Greater uncertainty was once again observed in the same question about emphysema diagnosis, where many responses were classified as “probably acceptable.”

Moreover, when comparing the two reconstruction kernels across both protocols and all questions, the distribution of the answers between the rating categories remained consistent, with no substantial differences observed. An exception is noted in the FD protocol for the question regarding the diagnosis of mediastinal inflammation, where the Standard kernel resulted in 19 per cent more images being rated as acceptable compared to the Lung kernel.

## Discussion

The present study investigated the impact of two different reconstruction kernels on AI-based CT image reconstruction. Specifically, the Standard kernel was compared with the newly introduced Lung kernel, both provided by GE Healthcare. The Lung kernel had not previously been applied with GE Healthcare’s DLIR algorithm at Sahlgrenska University Hospital. To further assess the influence of reconstruction kernels on image quality, two dose protocols were utilized: a FD protocol corresponding to standard clinical practice and a ULD protocol with radiation exposure comparable to that of a CXR taking place at the hospital [8].

So far, research comparing reconstruction kernels shows a preference for sharp kernels, especially for tasks such as assessing pulmonary nodules, because of their ability to enhance spatial resolution [30]. Smooth kernels, on the other hand, designed to improve tissue contrast while reducing noise, have shown superior performance in computer-aided detection systems and are increasingly utilized in clinical practice for evaluating lung parenchyma [31]. With the introduction of artificial intelligence and reconstruction algorithms based on deep learning, image quality has improved significantly, even at lower dose levels. Based on current knowledge, no studies to date have investigated the impact of different reconstruction kernels on CT images reconstructed using TrueFidelity. As a result, the comparison between different reconstruction kernels may now lead to different outcomes than those reported in earlier studies that used conventional reconstruction techniques.

Indeed, the present results showed that the spatial resolution achieved with TrueFidelity when using the Standard kernel was already sufficient for visualizing relevant anatomical details and performing routine diagnostic tasks, meaning that in some cases, sharper kernels might not offer much additional benefit. This observation was supported by the findings of Questions Q1, Q2, and Q4, that focused on the visualization of the major fissure and bronchi. In these cases, the Lung kernel had minimal impact, with no statistically significant differences compared to the Standard kernel. A similar pattern was observed in Questions Q7 and Q8, which assessed diagnostic acceptability for pulmonary nodules and fibrosis. However, it should be noted that for the ULD protocol, the observers mostly rated the images as “probably acceptable” or “unacceptable” for both kernels. The high proportion of non-acceptable images may explain the lack of statistically significant differences observed between the two kernels. In contrast, for the FD protocol, where the majority of images were regarded as acceptable for the evaluation of different pathologies, results are clearer. In half of the questions, the Standard kernel performed better. Specifically, the Standard kernel demonstrated superior performance for vascular structures, such as the pulmonary artery (Q3) and pulmonary vein (Q5), in both protocols. It also yielded higher ratings for the mediastinal lymph node (Q6) under the FD protocol. Additionally, for questions addressing emphysema (Q9) and mediastinal inflammation (Q10) under the FD protocol, the Standard kernel again showed better results. This is also illustrated in Fig. 5, where for both questions related to the Standard kernel, a higher number of images were rated as acceptable compared to those reconstructed with the Lung kernel.

The present findings suggest that the Standard kernel may offer a better balance between image sharpness and noise suppression when combined with TrueFidelity. Traditionally, high-resolution kernels have been favored for evaluating small or detailed structures like vessels or nodules due to their ability to enhance edge definition. However, deep learning-based reconstruction methods, such as TrueFidelity, appear to reduce the need for such kernels, as they already improve both resolution and noise characteristics. In the present study, the Standard kernel used with TrueFidelity not only preserved diagnostic quality but also outperformed the Lung kernel in several clinically relevant scenarios.

The only exception was observed in the evaluation of mediastinal inflammation (Q10) under the ULD protocol, where the Lung kernel statistically received significantly higher ratings. For this question the AUC was equal to 0.55, the lower bound of the CI slightly exceeded 0.5 (0.506) and the *P*-value was relatively close to .05 (.04). However, as the VGC analysis does not include corrections for multiple comparisons the results should be interpreted with caution. It is possible that this specific finding is due to a Type I error. Further studies need to be conducted to examine and confirm this observation.

Nevertheless, this study has several limitations that should be acknowledged. First, the relatively small sample size of 25 patients may limit the statistical power of our study and could have contributed to the lack of statistically significant differences observed between the two reconstruction kernels. Further studies need to be conducted including a larger number of cases to evaluate the performance of the reconstruction kernels in combination with deep learning reconstruction algorithms.

Except for the patients, the limited number of observers also plays a significant role. The results would have been more valid if more observers participated in the study since it would have been easier to generalize our conclusions. In this study, the observers were treated as fixed readers, meaning that data from all participating observers were included during resampling, and the findings are therefore applicable only to those individuals. This approach is recommended when the number of observers is small or when they may not adequately represent the wider population. Treating the observers as a fixed effect limits the statistical uncertainty to the specific observers involved in the study, rather than extending it to a broader group [32].

In addition, diagnostic acceptability varied across different dose levels and diagnostic questions, indicating that the optimal reconstruction kernel is influenced by both the imaging protocol and the specific clinical task. The results indicate that the ULD protocol makes it challenging for readers to discern differences between kernels, primarily due to the reduced radiation dose rather than equivalent diagnostic performance. Moreover, general image quality scores should be interpreted with caution, as the most suitable reconstruction method may differ depending on the presence and type of pathology. Future studies evaluating detection of pathologies would be beneficial to validate the results from the present study.

It should also be noted that all CT examinations included in this study were performed without the use of IV contrast media, a factor that directly impacts image quality. The final results might be different than the current ones if IV contrast was used in the examinations. Furthermore, the choice of using DLIR-H for image reconstruction was based on the findings of Svalkvist *et al*. [1], where DLIR-H was identified as the preferred setting when comparing TrueFidelity with Standard kernel to ASIR-V. However, it is important to acknowledge that alternative DLIR settings, such as low or medium strength, may potentially be more suitable when used in combination with the Lung kernel which is an aspect that has not yet been evaluated.

## Conclusion

In the present study, two different CT protocols reconstructed with the TrueFidelity (DLIR-H) algorithm from GE Healthcare were examined to assess the suitability of two different reconstruction kernels. For the FD protocol, the Standard kernel was rated higher than the Lung kernel for the majority of questions. For the ULD protocol, there were in general no statistically significant differences in ratings between the Standard and Lung kernel.
